# The Management of IgG4-Related Disease in Children: A Systematic Review

**DOI:** 10.3390/children12020213

**Published:** 2025-02-11

**Authors:** Evdoxia Sapountzi, Eleni P. Kotanidou, Vasiliki-Rengina Tsinopoulou, Lampros Fotis, Liana Fidani, Assimina Galli-Tsinopoulou

**Affiliations:** 1 Outpatient Rheumatology Unit, 2nd Department of Pediatrics, School of Medicine, Faculty of Health Sciences, Aristotle University of Thessaloniki, AHEPA University General Hospital, 54636 Thessaloniki, Greece; 2 2nd Department of Pediatrics, School of Medicine, Faculty of Health Sciences, Aristotle University of Thessaloniki, AHEPA University General Hospital, 54636 Thessaloniki, Greece; epkotanidou@auth.gr (E.P.K.); vitsinop@auth.gr (V.-R.T.); sfidani@auth.gr (L.F.); agalli@auth.gr (A.G.-T.); 3 Division of Pediatric Rheumatology, Department of Pediatrics, ATTIKON General Hospital, National and Kapodistrian University of Athens, 12462 Athens, Greece; lafotis@med.uoa.gr; 4 Laboratory of Genetics, School of Medicine, Faculty of Health Sciences, Aristotle University of Thessaloniki, 54124 Thessaloniki, Greece

**Keywords:** IgG4 disease, corticosteroids, rituximab, adalimumab, steroid-sparing agent, immunosuppressants

## Abstract

**Background/Objectives**: IgG4-related disease (IgG4-RD) is a multi-organ disease with greatly varying therapeutic approaches and a lack of specific treatment algorithms. This systematic review aimed to determine the therapeutic approaches for pediatric IgG4-RD in real-word practice. **Methods**: We searched PubMed and Google Scholar for articles on pediatric IgG4-RD cases published in English from 2012 to August 2024, focusing on treatments and outcomes. Study type, treatment(s), dose/regimen, age and sex, organ(s) involved, and treatment outcomes were manually extracted from each study. **Results**: Of the 219 studies identified, we analyzed 81 studies, including 114 pediatric IgG4-RD cases. Fifty-seven percent of patients suffered from multi-organ disease and required several treatment schemes. Around 75% received steroids, alone or in combination, regardless of the organ affected. The treatment outcomes were positive in most cases, although relapses occurred in approximately 30% of patients, usually upon steroid tapering. Other common therapeutic approaches included immunosuppressants, often used as steroid-sparing agents, with azathioprine and mycophenolate mofetil being the most common; surgery for localized disease; and biologics, mainly rituximab, used in more severe/refractory cases. Uncommon but effective therapies included adalimumab and ruxolitinib. Drug combinations seemed to be more efficacious than monotherapies across studies. Patients > 10 years old more frequently received aggressive approaches (surgery and rituximab) and more often experienced relapses. Relapse rates were higher among females. **Conclusions**: This review highlights the use of systemic steroids as an effective first-line treatment for pediatric IgG4-RD, but also underscores the use of non-steroid-based alternatives in combination with steroids or other immunosuppressants for the effective management of IgG4-RD.

## 1. Introduction

IgG4-related disease (IgG4-RD) is a relatively recent clinical entity that has emerged as a significant consideration in the field of pediatric medicine. IgG4-RD is characterized by unique histopathological features and commonly by multi-organ involvement, presenting a constellation of challenges, both diagnostic and therapeutic, particularly in pediatric patients. This immune-mediated condition, known for its fibro-inflammatory manifestations, remains an enigma due to its clinical overlap with various other autoimmune and inflammatory diseases. Since its first recognition as a distinct clinical entity in 2001 [1], international teams have made extensive efforts to align regarding its disease recognition, pathology, and patient management. Thus, the first consensus statement on the pathology of IgG4-RD was published in 2012, while an International Consensus Guidance statement on the treatment of IgG4-RD was issued in 2015 [2,3,4]. In 2019, the American College of Rheumatology and European League Against Rheumatism issued classification criteria for IgG4-RD in an effort to improve its disease recognition [5].

Pediatric cases of IgG4-RD are not only less common, but also display clinical presentations that differ markedly from those in adults [6]. This variance in symptomatology and disease progression underscores the importance of a focused inquiry into this unique cohort. Children are not merely small adults; their physiological and immunological systems are in a state of development that influences disease expression and response to treatment. Furthermore, the impact of IgG4-RD and its management on the growth and development of children presents an additional layer of complexity, warranting a thorough investigation.

According to the International Consensus Guidance, all patients with symptomatic, active IgG4-RD should be treated, with some requiring urgent and aggressive intervention to control this highly active disease in specific organs and avoid irreversible damage [4]. Such aggressive therapy may include combinations of corticosteroids at high doses, the use of mechanical devices (e.g., stents and catheters), or the use of biologics [4]. Untreated IgG4-RD may indeed cause irreversible organ damage and can be detrimental for patients [7]. However, evidence to guide the management of IgG4-RD is limited due to the rarity of the disease, especially among children. Systemic steroids serve as the primary treatment option in both adults and children [4], with most experts suggesting maintenance of the initial dosage for at least 2–4 weeks before gradual tapering and eventual discontinuation within 3–6 months of initiation. Importantly, 24–54% of patients relapse after steroid discontinuation and need to be retreated with steroids [8]. In these cases, steroid-sparing agents (SSAs) are the preferred option to avoid long-term steroid exposure. Among SSAs, rituximab, a monoclonal antibody targeting CD20, has shown great promise as a treatment strategy for IgG4-RD [9].

Despite this consensus guidance, treatment schemes and doses vary greatly in real-world clinical practice depending on geographical, epidemiological, and clinical factors, and especially on organ involvement. Although there is an abundance of data from adult patients, pediatric cases are scarce, and treatment approaches have not been captured in real-world scenarios. In this paper, we systematically review the treatment protocols used in pediatric IgG4-RD cases, aiming to uncover treatment patterns according to organ involvement and patients’ age and sex, as well as to highlight the most effective treatments by capturing treatment outcomes. By synthesizing the available data, this review offers a clearer understanding of IgG4-RD in the pediatric context, highlighting current treatment approaches and suggesting directions for future research, thus aiming to enhance the understanding of the management of IgG4-RD in children.

## 2. Materials and Methods

### 2.1. Search Strategy

The study design, protocol, and methods were based on the Preferred Reporting Items for Systematic reviews and Meta-Analyses (PRISMA) statement and registered in the Open Science Framework (OSF) database with the following Digital Object Identifier (DOI): https://doi.org/10.17605/OSF.IO/SY54A. The following search string was used to retrieve relevant articles published in PubMed and Google Scholar: ((IgG4-related disease) OR (IgG4-RD) OR (IgG4-related sclerosing disease) OR (immunoglobulin G4 related disease)) AND ((pediatric) OR (children) OR (juvenile) OR (adolescent) OR (childhood) OR (infant)) AND ((treatment) OR (corticosteroid) OR (cyclophosphamide) OR (tacrolimus) OR (cyclosporine) OR (methotrexate) OR (azathioprine) OR (prednisone) OR (prednisolone) OR (tocilizumab) OR (rituximab) OR (steroid) OR (belimumab) OR (mycophenolate) OR (surgery) OR (plasmapheresis) OR (outcomes) OR (outcome measures)). The first consensus statement regarding the definition of the IgG4-RD entity and its management was published in 2012. Therefore, we limited our search to articles published from 2012 onwards (search date: 22 August 2024).

The inclusion criteria were studies in English involving pediatric patients (<18 years old) with a probable or definite IgG4-RD diagnosis, in which the regimen used to treat the patients (including drug therapy or surgical intervention) was detailed. The exclusion criteria were randomized placebo-controlled studies, case–control studies, and studies in which the selection criteria for patients focused on a specific treatment, as our goal was to capture the real-word treatment regimens used in pediatric patients with IgG4-RD.

### 2.2. Data Collection and Handling

The following information was extracted from each study: type of study, number of patients, patients’ age and sex, treatment used, treatment dose and regimen, organ involvement, and treatment outcomes. These data were extracted manually from each study. If the age of patients was reported in months, it was converted to years by dividing by 12.

The mean age of patients and the distribution of patients according to sex were calculated using Microsoft Excel. This was also used to calculate the number of patients for each treatment, the number of patients with a particular organ involvement, and the number of patients with a positive or negative treatment outcome. Diagrams were generated using Microsoft Excel. A pictorial representation of the key study findings was created using Microsoft PowerPoint.

A review management tool was used to assess eligible studies, and a prototype data extraction form was also created to categorize all extracted data. The selection process and data extraction process were performed independently by two investigators (E.S. and L.F.; screening and inclusion based on eligibility criteria). All disagreements were resolved by A.G.-T.

### 2.3. Risk of Bias Assessment

Since most of the selected studies were case reports or case series, we employed the modified Newcastle–Ottawa Scale to rate the risk of bias for each study. Accordingly, the studies received 0–4 points for the selection of cases, which included 1 point for the representativeness of the cases, i.e., clearly defined cases and cases typical of IgG4-RD; 1 point for the selection of controls, if applicable; 1 point for ascertainment of exposure, i.e., well-documented treatment or risk factors; and 1 point if the outcome of interest was not present at the start of the study. Further, the studies received 0–2 points for comparability, i.e., if they controlled for the major confounding factor (1 point) or additional factors (1 point). The outcome assessment was rated with 0–3 points, 1 for a clear definition and measurement of the outcome, 1 for a sufficient follow-up time to capture the outcome (e.g., >12 months), and 1 if more than 80% of the patients were followed up or if an explanation was provided for patients lost to follow-up.

## 3. Results

**Figure 1:**
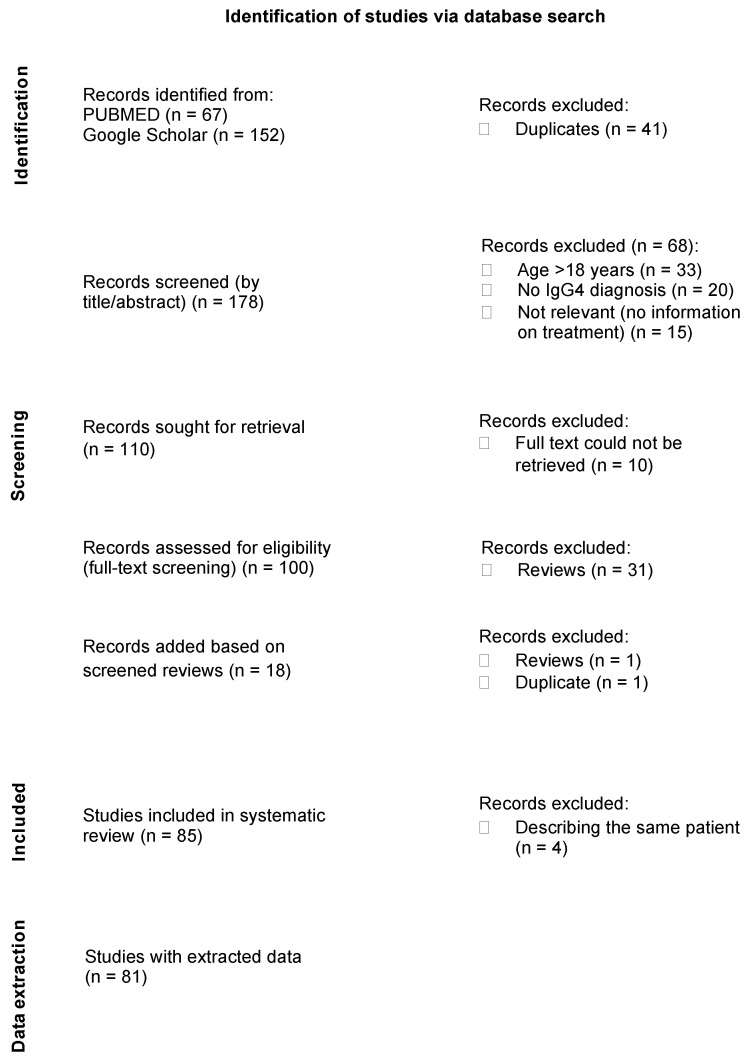
Study selection flow chart.

In total, 219 articles were identified via a PubMed and Google Scholar search. After removing 41 duplicates, 178 articles were screened further for relevance via titles and abstracts. Sixty-eight articles were excluded because they did not fulfill one of the inclusion criteria, and ten articles were excluded because we could not retrieve the full text. Thirty-one reviews, including systematic reviews, were also screened to retrieve additional articles, but were otherwise excluded from the final list of analyzed articles. Eighteen articles were found based on this screening, one of which was a review and one was a duplicate, so both were excluded. Thus, 85 relevant studies were included in our systematic review. Of these, four more studies were excluded as they described the same patient, as deducted by the list of authors, title, patient’s age, clinical characteristics, treatments, and treatment outcomes. Finally, the information from 81 studies was extracted and analyzed for treatment patterns. The study flow chart is shown in Figure 1. The vast majority of the included analyzed studies were case reports.

### 3.1. Patients

With this systematic literature review, we identified 114 pediatric cases of IgG-RD. The patients’ age ranged from 15 months to 17.5 years (mean 11.5 years; median 12 years), and there were 40 patients younger than 10 years. There was an equal representation of girls and boys in the included studies (58 girls, 55 boys, and 1 not reported). IgG4-RD was commonly diagnosed on the basis of clinical symptoms, imaging, and biopsy results, as well as according to histological evidence of IgG4-positive plasma cell infiltration.

### 3.2. Organ Manifestation

The cases described in this study showed a spectrum of different organ manifestations. The lymph nodes were the most common organ involved, in 34 of 114 patients (29.8%), often in association with other organs, most commonly including the liver in 10, eye/orbit in 7, lung in 6, and pancreas in 4 patients.

The orbit, including the lacrimal glands, was the next most frequently involved site in the collected cases, observed in 33 of 114 patients (28.9%). Patients with orbital involvement often presented with proptosis, lacrimal gland enlargement, and eyelid swelling, as well as orbital masses. Some also experienced vision changes. Notably, 31 of 33 patients with orbital involvement were girls.

The biliary system, including the liver, was involved in 22 of 114 patients (19.3%), often manifesting as sclerosing cholangitis, hepatosplenomegaly, or intrahepatic duct strictures. The pancreas was involved in 15 of 114 patients (13.2%), typically presenting as autoimmune pancreatitis or a pancreatic mass. In total, 10 of the patients with pancreatic involvement also had biliary system involvement (liver n = 9, bile ducts n = 6, and liver + bile ducts n = 3), while 6 patients also had lymphadenopathy.

The lungs were involved in 18 of 114 patients (15.8%). These patients presented with pleural effusion, inflammatory pseudotumor, mediastinal masses, or bronchiectasis, and often experienced dyspnea, recurrent infections, or hemoptysis.

The CNS, gastrointestinal tract, and kidneys were affected in 10 patients each.

**Figure 2:**
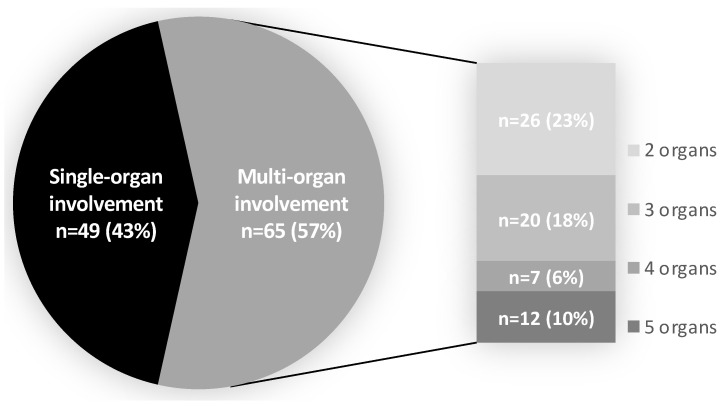
Organ involvement. Multi-organ involvement was considered if 2 or more organs were involved. The number of patients and percentage having 2, 3, 4, or 5 organs involved are shown on the right of the pie graph.

IgG4-RD involved a single organ in 49 patients (43%), while the majority of patients (n = 65, 57%) suffered from multi-organ disease (≥2 organs involved) (Figure 2), in accordance with the multi-system involvement observed in IgG4-RD.

### 3.3. Therapeutic Approaches

**Figure 3:**
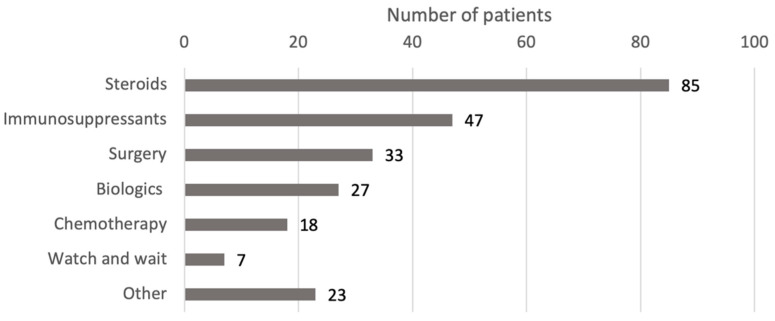
Treatment modalities by drug class. Steroids include prednisone, prednisolone, methylprednisolone, corticosteroids, steroids, deflazacort, betamethasone, and budesonide. Surgery includes excisions, debulking, resections, ectomies, stent, transplant, and the Whipple procedure. Biologics include rituximab, adalimumab, immunoglobulins, and infliximab. Chemotherapy includes methotrexate, cyclophosphamide, and 6-mercaptopurine. Immunosuppressants include azathioprine, MMF, sirolimus, and cyclosporine. Immunomodulators include tacrolimus, IFNb-1b, and teriflunomide. Antibiotics include ceftriaxone, cefixime, azithromycin, and trimethoprim/sulfamethoxazole. Abbreviations: IFN, interferon; MMF, mycophenolate mofetil.

**Figure 4:**
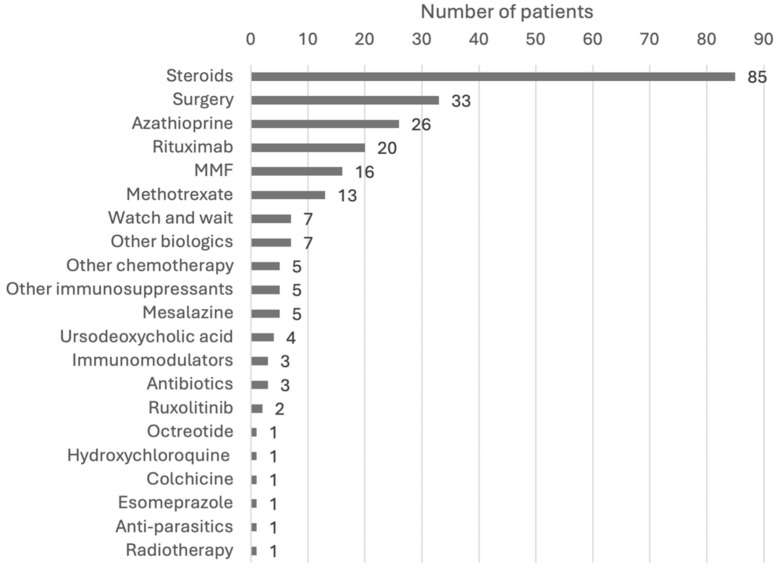
Breakdown of treatment modalities. Steroids include prednisone, prednisolone, methylprednisolone, corticosteroids, steroids, deflazacort, betamethasone, and budesonide. Surgery includes excisions, debulking, resections, ectomies, stent, transplant, and the Whipple procedure. Other biologics include adalimumab, immunoglobulins, and infliximab. Other chemotherapy includes cyclophosphamide, and 6-mercaptopurine. Other immunosuppressants include sirolimus and cyclosporine. Immunomodulators include tacrolimus, IFNb-1b, and teriflunomide. Antibiotics include ceftriaxone, cefixime, azithromycin, and trimethoprim/sulfamethoxazole. Abbreviations: IFN, interferon; MMF, mycophenolate mofetil.

The treatments used for IgG4-RD varied greatly, reflecting the heterogeneity of organ involvement and disease severity. The most commonly used therapeutic approaches were steroids, immunosuppressants, surgery, and biologics (Figure 3). In several cases, these modalities were used in combination, either simultaneously or sequentially, based on the patient’s response. Figure 4 shows a more detailed diagram of treatment frequencies.

#### 3.3.1. Steroids

Steroids were the cornerstone of treatment, used in 85 of 115 patients (73.9%), either as a monotherapy or in combination, and usually as a first-line therapy (Table 1). Prednisolone or prednisone was the most commonly administered steroid (in 63 of 85 patients). In the studies that mentioned the steroid dose, these doses ranged from 0.5 to 2 mg/kg/day, with tapering protocols varying between 4 weeks and 6 months. In most cases, steroid therapy resulted in symptom relief and disease control; however, relapses were reported in almost one-third of cases (23 of 85, 27.1%), often upon steroid tapering/discontinuation. In these cases, retreatment with steroids or the use of immunosuppressants was chosen. High-dose steroids such as pulse intravenous methylprednisolone (IVMP) were mostly used following non-response/partial response to a previous treatment or following a relapse. Of six cases with orbital involvement treated with pulse IVMP, the treatment was effective in four cases, leading to minimal clinical improvement in one case [10], and its discontinuation was followed by a relapse in three cases [11,12,13]. No clinical improvement with IVMP was seen in one case of multi-organ disease (five organs involved; [14]), although other cases with multi-organ involvement were more responsive to IVMP.

#### 3.3.2. Immunosuppressants

Immunosuppressants were used in 47 of 114 patients (41.2%).

Azathioprine was the most common immunosuppressant used, administered to 26 patients (22.8%) as an SSA. It was used at doses ranging from 0.5 to 2.5 mg/kg/day and commonly in combination with steroids (22 of 26 patients). This combination was effective in maintaining remission and reducing steroid dependency or in achieving response after a relapse. In some patients, however, the combination or azathioprine monotherapy was not effective [89] or induced an initial response, followed by relapse [40,61,64,77].

Mycophenolate mofetil (MMF), the second most common immunosuppressant used, was administered to 16 patients (14.0%). MMF was often employed after initial response to steroids, particularly in cases of orbital involvement. The combination of MMF and steroids provided effective disease control in patients with orbital pseudotumor, proptosis, and lacrimal gland involvement, with minimal relapses [26,48,56,73,75,80,84]. No response to MMF was noted in two patients, one with lymph node and eye involvement [23] and one with orbital involvement [17], both of whom were also not responsive to prednisone and methotrexate. A poor response was noted in a patient with biceps muscle, lymph node, liver, and spleen involvement who had relapsed upon prednisolone tapering [68]. In these two patients, remission was achieved with rituximab.

Other less frequently used immunosuppressants included sirolimus, effective for IgG4-related lymphadenopathy in one patient [55] and for systemic IgG4-RD in combination with rituximab in another patient [53], and cyclosporine, administered alone or in combination with steroids in three patients but showing efficacy only in one with IgG4-related uveitis [64]. In the other two patients, no response and/or relapse was noted [23,73].

#### 3.3.3. Surgical Intervention

Surgery was performed in 33 (28.9%) patients. Surgery was primarily utilized for localized disease, such as orbital pseudotumors, glandular involvement, or soft-tissue masses, or for diagnostic purposes. Surgical intervention often led to complete remission without further pharmacologic intervention. Stent placement, bone marrow transplant, and the Whipple procedure were applied to one patient each.

#### 3.3.4. Biologics

Biologics were administered to 27 patients (23.7%), with rituximab being the most common biologic used (20 patients, 10 females), especially in those with recurrent or resistant IgG4-RD [10,14,23,39,49,57,68]. Most patients treated with rituximab had multi-organ involvement, while all 10 girls who received this therapy had orbital involvement. Rituximab was used as the only therapy in one patient with bilateral orbit and lung involvement with a marked improvement in symptoms after 9 months of follow-up [21]. In some patients, it was used in combination with steroids and immunosuppressants. Most patients who received rituximab achieved complete remission or stable disease, except one patient, who showed resistance to treatment [12], and two patients who showed only partial response [49,89]. One of these latter two patients relapsed after the partial response and was retreated and maintained with rituximab. Similarly, another patient relapsed after the first course of rituximab treatment, but showed the complete resolution of symptoms after a second course, this time in combination with steroids [39]. Relapse on rituximab also occurred in one patient with pericardium and lung involvement who showed an initial improvement in symptoms with rituximab, but relapsed and died 6 months later [77].

Other biologics administered included adalimumab, infliximab, and immunoglobulins. Adalimumab, given to three patients (two girls), led to a complete and rapid resolution of symptoms in all [44,45,61], and remission was maintained for 1 year post-treatment [45,61]. One of these patients, a 16-year-old girl diagnosed with IgG4-sclerosing disease, had received infliximab prior to adalimumab with no response [61]. In a 9-year-old girl with isolated orbit involvement, adalimumab was given off-label at 40 mg twice weekly subcutaneously, leading to a complete response [45].

#### 3.3.5. Chemotherapy

Of the analyzed cases, 18 (15.8%) were treated with chemotherapy agents, with methotrexate being the most frequent one (in 13 patients, 7 girls). All patients who received methotrexate had also received corticosteroids previously or were started on methotrexate while on steroid treatment. Cyclophosphamide was given to four patients (all girls with eye/orbit involvement) in combination with steroids, leading to a partial or complete response, as well as the maintenance of stable disease. Six-mercaptopurine was administered to an 11-year-old female with autoimmune pancreatitis type 1, leading to symptom control and no relapse for up to 5 years of follow-up [36].

#### 3.3.6. Other Treatments

Other treatments used alone, in combination with, or following steroids included mesalazine (n = 5); ursodeoxycholic acid (UDCA; n = 4); the JAK inhibitor ruxolitinib (n = 2); different immunomodulators such as tacrolimus, IFNb-1b, and teriflunomide (n = 1 each); octreotide (n = 1); and radiotherapy (n = 1).

Patients who received mesalazine suffered from ulcerative colitis or inflammatory bowel disease, in agreement with the indications approved for this drug. These patients had multi-organ IgG4-RD (three or more organs involved), involving the pancreas in most cases. Mesalazine was used as a maintenance therapy in three of the patients and as a first-line therapy in two. It led to a complete resolution of symptoms in one patient, in whom it was administered together with IVMP [15].

All four patients that received UDCA suffered from sclerosing cholangitis and had from two to four more organs involved other than the liver. When used as a monotherapy, UDCA induced no or only partial remission [15,20], whereas a complete response or significant improvement was noted when it was given in combination with steroids [20,43]. The patient who showed significant improvement with UDCA had been initially treated with prednisolone, and UDCA was added one month later together with mesalazine and azathioprine [43], indicating that this multi-drug approach may be more efficient than the monotherapy options.

Tacrolimus and IFNb-1b, administered in combination with other regimens, led to clinical improvement or stable disease, whereas teriflunomide was not effective in managing lymph node enlargement in a patient with multi-organ IgG-RD [73].

Two patients were treated with the JAK inhibitor ruxolitinib, which was effective in managing localized disease [53]. Octreotide, a somatostatin analog, was used in a 16-year-old boy with pleural effusion, a mediastinal mass, and mesenteric lymph node involvement. The patient had shown an initial response to prednisone, but relapsed after tapering. Octreotide was administered following a lack of response to prednisone plus azathioprine treatment; however, it also failed to reduce the pleural effusion and mediastinal mass. Surgical intervention was finally needed for this patient, leading to improvement in the pleural effusion [40].

Radiotherapy was applied in a 9.3-year-old girl with orbital swelling and proptosis and fifth cranial nerve involvement. Two courses of radiotherapy were needed in this case for achieving remission after no response to steroids, rituximab, and azathioprine [12].

#### 3.3.7. Watch-and-Wait Strategy

Among the identified cases, there were seven patients that did not receive any treatment for their IgG4-RD. Instead, the selected approach was to watch and wait for the symptoms to resolve. Four of these patients had lymph node involvement only [55,58], one had orbit involvement only [12], one had hepatosplenomegaly in addition to his lymphadenopathy [58], and one patient had kidney and salivary grand involvement in addition to his lymphadenopathy [18]. The latter patient was subjected to renal biopsy, which confirmed the diagnosis of IgG4-RD. Seven months after diagnosis, PET/CT showed the worsening of kidney uptake, which would mandate some treatment, although this was not mentioned by the study authors [18]. For the five patients with lymphadenopathy, the watch-and-wait strategy proved beneficial, as there was no clinical progression noted during follow-up (0.2 up to 8 years). For the patient with orbital involvement, despite an initial spontaneous regression of symptoms, a relapse occurred 38 months after diagnosis, which was treated with pulse methylprednisolone, followed by prednisolone for 1.5 months [12].

### 3.4. Age and Sex

Since most studies analyzed were cases reports or case series, there was no control for confounders such as age or sex. Keeping this in mind, our analysis showed potential trends in treatment approaches and outcomes. For example, more aggressive approaches such as surgery and rituximab therapy were used more frequently in older children (>10 years) than in younger ones (≤10 years). Specifically, 16 and 10 patients aged more than 10 years were subjected to surgery and received rituximab, respectively, versus only 11 and 4 patients aged 10 years or less, respectively. Such aggressive approaches seemed to be justified, as older children were also more likely to experience a relapse (n = 19 aged >10 years versus n = 11 aged ≤10 years). The relapse rates were also higher among females (n = 18) than among males (n = 12), although it is not clear if this difference is clinically meaningful, as sex has not been reported as a predictor of relapse in IgG4-RD [90,91]

## 4. Discussion

**Figure 5:**
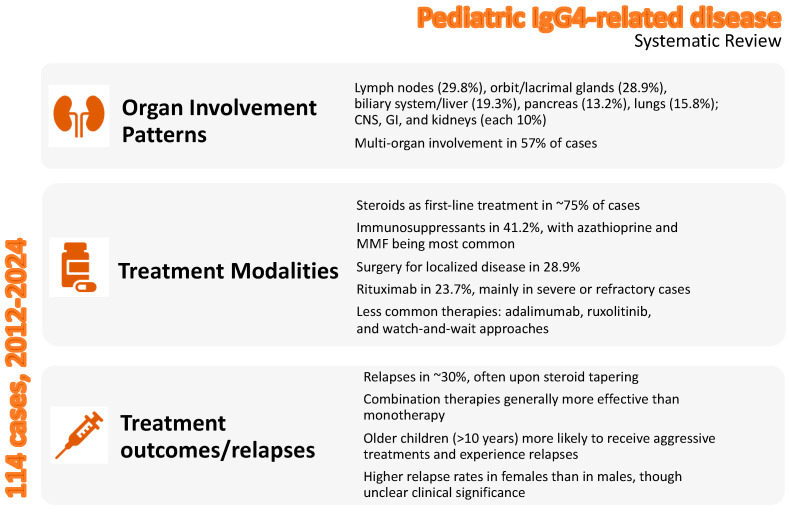
Overview of the key findings of the systematic review in pediatric IgG4-related disease.

The primary outcome of this systematic review was to uncover the treatment approaches undertaken in real-world clinical practice for pediatric IgG4-RD. By synthesizing data from 81 studies encompassing 114 pediatric patients, this review provides critical insights into the management of this rare and challenging condition and highlights significant variability in its clinical presentation, organ involvement, and therapeutic strategies. The key findings of this systematic review are summarized in Figure 5.

IgG4-RD is a rare fibroinflammatory condition generally occurring in middle-aged patients, and is even more uncommon in children. Our study confirms the rarity of IgG4-RD in the pediatric population, as we identified only 114 cases occurring between 2012 and 2024, in a span of 12 years. In their systematic review, Karim et al. [92] identified 25 pediatric patients with IgG4-RD over a 5-year span (2010–2015). Considering the 3-year overlap between ours and their systematic review, some of the identified cases overlap. In a descriptive review, Hara et al. [6] reported 135 IgG4-RD cases, many of which also overlap with the cases identified in our study. The authors identified more cases than we did, because they included patients up to the age of 25 years. Considering these two reviews and our findings, less than 150 pediatric cases have been reported since 2010. Nevertheless, considering the diagnostic challenges of this condition, pediatricians should remain alert so as to promptly recognize the disease and provide effective treatments for patients.

IgG4-RD is thought to be more prevalent in men than in women [93]. Among the limited number (N = 25) of pediatric patients reported by Karim et al., sex prevalence followed the opposite trend, with girls being more affected than boys. In contrast, among the 114 identified pediatric IgG4-RD cases in our study, no such trend was found, and both sexes were equally represented, arguing against a sex preference of the disease. The same conclusion was reached by Hara et al. [6]. Further, we found that the pediatric cases demonstrated distinct patterns of organ involvement compared to those in adults. The lymph nodes, orbit, and biliary system were the top three affected sites in our cohort. In an adult cohort of 82 patients, the most frequently affected organs were the lymph nodes (50.6%), pancreas (38.7%), and salivary glands (35.6%) [94]. In a much larger adult cohort, pancreatic, hepatic, and biliary involvement were the most prevalent (in 31%) [95]. These distinct patterns underscore the importance of tailoring diagnostic and therapeutic strategies to pediatric patients, as their disease manifestations and responses to treatment may differ substantially.

In our study, we did not collect information on disease severity from the included studies, because most of them lacked objective measures of disease activity such as the IgG4-RD responder index (RI). Our analysis showed 57% of pediatric patients suffering from multi-organ disease, with 16% having 4–5 organs involved. Serum IgG4 levels, IgG4-RD RI scores, the number of organs involved, and eosinophil count have been reported to reflect IgG4-RD disease activity [91]. However, disease severity may not be uniform across all organs, and some organs may even be asymptomatic [96]. Since disease severity across the involved organs is expected to influence treatment strategies, future studies are warranted to address this point.

Regarding therapeutic approaches, our findings are consistent with the existing literature, which identifies systemic corticosteroids as the cornerstone of IgG4-RD treatment, administered in almost 75% of the analyzed cases. A previous systematic review including 62 studies, encompassing more than 3000 adult patients, also reported glucocorticoid-based regimens in 75% [97]. The use of prednisolone and prednisone as a first-line therapy in most of the analyzed cases of our study led to high rates of symptom relief and disease control. The used doses and tapering protocols are consistent with those reported in the literature for adults, as well as with the 2015 International Consensus Guidance statement for IgG4-RD management [4].

Relapses were commonly seen with steroid monotherapy upon tapering or discontinuation, and still occurred even with the addition of immunosuppressive agents like azathioprine, indicating that some patients may require prolonged or more aggressive therapy. The observed relapse rate of 27.1% after steroid treatment is within the range of that reported in adults [8]. These results reinforce the need for adjunctive or maintenance therapies to mitigate steroid dependency. A high rate of multi-organ involvement across the included cases might explain the observed relapse rate, in line with previous studies [91].

Most included studies did not report side effects with the use of steroids. However, the potential adverse effects associated with long-term steroid use in children, including swelling, weight gain, hair growth, acne, growth delay, and Cushingoid features, should not be overlooked. Among the analyzed cases in our review, there was one case who developed Cushing syndrome after long-term steroid therapy [37]. The risk of side effects depends on the dose used (low dose < 10 mg/day of prednisone, medium dose 10–20 mg/day, and high dose >20 mg/day), type of steroid (long-acting vs short-acting), length of treatment (long-term treatment > 3 months), and other medical problems [98]. Although information on the used steroid doses and duration was not included in several studies, in those that provided this information, the treatment duration ranged from weeks to up to 2 years, while the doses ranged from 0.5 to 2 mg/kg/day, corresponding to low-to-medium dose levels. This might explain the lack of reported side effects. Only two studies reported a rather high dose (up to 25 mg/day [23] and 50 mg/day [30]) used for a very long period (2 years [23] and 9 months [30]), but reported no side effects. Our review suggests that, in real-world practice, physicians seriously consider the long-term effects of steroids in children and try to maintain low doses when treatment is required for longer periods or try to shorten treatment duration when high doses are needed for disease control. The use of SSAs and biologics in the included cases might also have been helpful in mitigating the risk of corticosteroid-associated side effects, as well as aiding in improving disease management.

SSAs such as azathioprine and MMF were the two most commonly used immunosuppressants in the analyzed cohort and were generally effective in maintaining remission or reducing steroid dependence, in agreement with efficacy study results in adult patients [97,99]. However, cases of non-responsiveness or relapse suggest the potential insufficiency of immunosuppressive therapy, underscoring the need for further studies to identify predictive markers of response to these agents. Similarly, rituximab, the most frequently used biologic, demonstrated significant efficacy in patients with multi-organ disease or steroid-refractory cases. Cases where rituximab was used in combination with steroids showed less frequent relapses, suggesting that it may be more effective in preventing recurrence when used in combination with steroids or other immunosuppressants. In agreement, rituximab has been shown to increase remission and reduce relapse rates in IgG4-RD [100] and has been associated with successful outcomes in various organ systems, showing efficacy in cases where glucocorticoids and other immunosuppressive agents fail [101]. Nevertheless, partial responses, relapses, and resistance (in one case) were observed in our analysis even with rituximab, suggesting the potential role of combination therapy, individualized dosing regimens, or repeat therapy protocols to achieve better outcomes.

Our analysis indicated some organ-specific responses to treatment, suggesting that individualized regimens should be tailored to the affected sites. For example, the combination of steroids and MMF was highly effective in managing orbital involvement, including pseudotumors and proptosis, while rituximab was effective for extensive multi-organ disease. Multimodal approaches such as surgery and biologics were required in cases with biliary system and pancreas involvement. Other therapies such as tacrolimus and teriflunomide showed a limited effectiveness in certain organ systems, suggesting that these agents may have a niche role, requiring further evaluation in targeted studies. In contrast, ruxolitinib was effective in localized cases, in agreement with the potential benefit of JAK inhibitors in IgG4-RD [102].

### 4.1. Limitations

The study results should be interpreted considering certain limitations. First, the vast majority of studies included in this systematic review were case reports or case series, which typically focus on specific, well-defined patient cases and individual patient outcomes and, hence, do not provide broader population-level data, which limits the generalizability of such findings. Moreover, case reports inherently lack a comparison between treatment groups or a control group and, thus, cannot adequately control for confounding factors. Hence, we cannot exclude the possibility of selection bias, and it is difficult to draw definitive conclusions about treatment efficacy beyond individual cases. Second, while the included studies tracked treatment outcomes, many only focused on the short-term efficacy and did not address long-term efficacy, which further limits the conclusions that can be drawn. Thus, although the treatment outcomes were generally positive, the findings must be interpreted with caution due to the limitations of the study designs. Finally, it is possible that some of the reported cases were mimickers of IgG4-RD and not true IgG4-RD, since we relied on the diagnosis made by the authors of each study, which was not necessarily based on the currently accepted diagnostic criteria for the disease. However, even if some cases were not true IgG4-RD, this argues even more for the rare manifestation of the disease in pediatrics. Despite the above limitations, our study captured real-world practices in IgG4-RD management, without attempting to generate any statistical results other than population frequencies.

### 4.2. Conclusions and Future Perspectives

The findings of this systematic review underscore the importance of a multidisciplinary approach in managing pediatric IgG4-RD. While systemic steroids remain the first-line treatment, prolonged use can lead to significant side effects, especially in children (e.g., growth delay and bone thinning). Therefore, future research should focus on developing strategies to reduce steroid dependence. Immunosuppressive drugs like azathioprine, MMF, and methotrexate are already used in some cases. Investigating their efficacy in combination with or as alternatives to steroids in children will be important for reducing side effects. Further, adjunctive therapies should be considered early in patients at high risk of relapse or those with severe multi-organ involvement. The emergence of biologics like rituximab offers a promising alternative, particularly for refractory cases, but long-term safety and efficacy data are needed. Other biologics such as infliximab and adalimumab, both of which target tumor necrosis factor-alpha, have been used in refractory cases of autoimmune diseases. Research is needed to assess their effectiveness in pediatric IgG4-RD, particularly in cases with high disease activity or organ involvement. Moreover, the potential role of emerging therapies, such as JAK inhibitors (ruxolitinib) and targeted biologics, should be explored in larger pediatric cohorts. Ruxolitinib has shown potential in managing inflammatory conditions and, thus, investigating its role in pediatric IgG4-RD could provide an option for steroid-sparing therapy. Importantly, our review highlights the need to develop strategies to prevent relapses, including understanding the role of maintenance therapies and developing biomarkers or advanced imaging techniques. for relapse prediction, which could allow for timely interventions, thus reducing long-term damage. Considering the heterogeneity of IgG4-RD, personalized treatment plans based on the child’s specific disease manifestation will be crucial. Research into identifying genetic or immunological markers that predict response to therapy could help clinicians to choose the most effective treatment. The development of pediatric-specific treatment guidelines is critical to standardize care and improve outcomes. Global research collaborations across centers and countries will help to accumulate more data on the efficacy of various treatments and enable the development of evidence-based guidelines for treating pediatric IgG4-RD. This systematic review provides a basis towards this direction, additionally to informing physicians of current practices in pediatric IgG4-RD.

## Figures and Tables

**Table 1:** Identified pediatric cases of IgG4-related disease.

Study Type	N(P)	Treatment	Dose/Duration	Age	Sex	Organ Involvement	n(O)	Diagnosis	Treatment Outcome	Citation
Case report	1	UDCA; IVMP plus oral mesalazine	UDCA: 15 mg/kg; IVMP 1.5 mg/kg/day, tapered to 4 mg/day over 10 weeks, plus mesalazine 60 mg/kg/day	7	F	Pancreas (AIP 1), liver (sclerosing cholangitis), lacrimal glands, intestine (IBD)	4	IgG4-RD	No response to USDA. Complete resolution of pancreatic enlargement with prednisone/mesalazine. Normalization of IgG4 and inflammatory markers, no relapse during 12 months of follow up	[15]
Case report	1	Corticosteroids; surgery (tumor removal)	Steroids: DNS, 6 months	12	M	Lung (IPT)	1	IgG4-RD pulmonary IPT	Symptom improvement with steroids, complete remission after surgery	[16]
Case report	1	OP; MTX	OP: DNS, discontinued after 1.5 months. Restarted upon relapse, tapered. MTX: DNS; added at 2nd month as an SSA	14	F	Orbital involvement (right eye)	1	IgG4-ROD	Initial response to OP but relapse 1 week after discontinuation. Good control of symptoms after OP restart. No relapse at the 4-month follow up	[17]
Case report	1	Prednisolone, MTX, MMF; pulse MP plus cyclophosphamide	Prednisolone, MTX, MMF: DNS; discontinued due to lack of response. Pulse MP plus cyclophosphamide: DNS	9	F	Orbital involvement (right eye)	1	IgG4-ROD	No response to prednisolone, MTX, or MMF. Stable disease after pulse MP plus cyclophosphamide up to the 2-year follow up	[17]
Case report	1	No treatment, later renal biopsy	NA	17	F	Kidneys (tubulointerstitial nephritis), lymph nodes, salivary glands	3	IgG4-RD	Diagnosis of IgG4-RD following biopsy; no systemic treatment introduced. Worsening of kidney uptake by PET/CT after 7 months; no further outcomes reported	[18]
Case report	1	Prednisolone	DNS	7	M	Pancreas (AIP), cervical lymph nodes, liver (sclerosing cholangitis)	3	IgG4-RD	Sclerosis cholangitis occurred 1.5 year after prednisolone treatment	[19]
Case report	1	UDCA; corticosteroids; corticosteroids plus AZA	DNS; AZA started 1.5 year after start of corticosteroids	14	M	Liver (hepatomegaly), pancreas, biliary ducts, lymph nodes; ulcerative colitis	5	IgG4-RD/AIP	Partial improvement with UDCA, later relapse treated with prednisolone, and AZA added 1.5 years later due to frequent relapses	[20]
Case report	1	Prednisolone, AZA, tacrolimus, infliximab, MTX	DNS	11	F	Liver (hepatomegaly), bilateral cervical and inguinal lymph nodes, pancreas (mass), biliary ducts, colitis	5	IgG4-RD/AIP	Initial improvement with corticosteroids not sustained; relapse on AZA treated with multiple immunosuppressants, resulting in complete resolution of gastrointestinal disease and improvement in pancreatic mass; persisting changes in biliary ducts	[20]
Case report	1	Steroids and UDCA, added AZA	DNS	7	M	Pancreas, liver (chronic cholangitis), lymph nodes	3	IgG4-RD/AIP	Complete resolution of pancreatic enlargement. Patient followed for IBD	[20]
Case series	1	Rituximab	DNS	11	F	Bilateral orbit, lungs (nodules)	2	IgG4-RD	Marked improvement in swelling after 9-month follow-up	[21]
Case series	1	High-dose systemic steroids	DNS	15	F	Right orbit	1	IgG4-RD	Symptom improvement, no worsening of swelling after 16-month follow-up	[21]
Case series	1	High-dose systemic steroids, resection	DNS, steroids tapered following resection of eye swelling/mass	10	F	Right orbit (swelling, mass)	1	IgG4-RD	No recurrence after 32-month follow-up	[21]
Case series	1	Neck mass resection	NA	11	M	Extrathyroidal neck soft tissue	1	IgG4-RD	Mild persistent lymphadenopathy, otherwise asymptomatic after 11-month follow-up	[21]
Case report	1	Surgical resection; prednisolone, added rituximab; desmopressin for diabetes insipidus	Prednisolone (1 mg/kg/day for 3.5 weeks, tapered over 6 months), Rituximab added 1 month after prednisolone (1000 mg IV, 2 doses, 3 weeks apart)	14	F	Pituitary (mass, hypophysitis)	1	Isolated IgG4-related hypophysitis	Significant improvement, sustained remission after 20-month post-resection; patient lost to follow-up	[22]
Case report	1	Prednisone; MTX; cyclosporine; MMF, rituximab plus prednisone	Prednisone: up to 25 mg/day for 24 months; MTX: 15 mg/week; cyclosporine: 4 mg/kg/day; MMF: 2 g/day, stopped after scleritis relapse; rituximab: 1 g, IV, twice, 2 weeks apart plus prednisone 10 mg/day	17	M	Retroperitoneal lymph node, eye (scleritis)	2	IgG4-RD	Relapses with most treatments; improvement of scleritis and normal serum inflammatory biomarkers with rituximab plus prednisone	[23]
Case report	1	Surgery (excision of rectovesical pouch mass)	NA	9	M	Rectovesical pouch	1	IgG4-RD	Complete remission after mass excision, asymptomatic after 12-month follow-up	[24]
Case report	1	Prednisone	Prednisone: 0.6 mg/kg/day (20 mg/day) for 1 week, tapered over 6 months and stopped	9	M	Lymph nodes, liver, spleen, thalamus, lungs	5	Probable IgG4-RD	Significant improvement in eosinophil count and IgG4 levels, asymptomatic at last follow-up (about 1 year after treatment start)	[25]
Case report	1	IVMP, rituximab; MMF	IVMP: DNS, tapered; rituximab: DNS; MMF for maintenance (DNS)	10	F	Lacrimal glands, salivary glands, submandibular glands, lymph nodes	4	IgG4-RD	Significant improvement, remission maintained with MMF	[26]
Case report	1	Thoracoscopic partial resection; prednisone	Prednisone: 2 mg/kg, tapered over 6 months	1.8	F	Lungs (recurrent infections, bronchial compression), mediastinal mass, lymph nodes	3	IgG4-RD	Worsening of symptoms and mass size after resection. Significant improvement and mass reduced after prednisone treatment; stable for 12 months after stopping therapy	[27]
Case report	1	Desmopressin, surgical resection; steroid replacement	Desmopressin: 0.05 mg/BID; steroid replacement type not reported	16	F	Pituitary gland, suprasellar mass compressing optic chiasm	2	IgG4-related hypophysitis	Significant improvement after lesion resection, continued on steroid replacement and desmopressin; lost to follow-up	[28]
Case report	1	Steroids, subcutaneous MTX	MTX: 15 mg/m^2^/week plus weaning course of steroids	9	F	Orbit (right upper eyelid swelling, intra-orbital mass), right cheek	2	Provisional diagnosis of IgG4-ROD	Very good response	[29]
Case report	1	Pulse IVMP, cyclophosphamide; OP plus rituximab as maintenance	Pulse IVMP: 20 mg/kg for 5 days; added cyclophosphamide: 500 mg/m^2^/month as pulse, stopped at 6 months. Prednisolone: 10 mg/day plus rituximab 375 mg/m^2^/week for 4 weeks as maintenance and repeated 6 months after first infusion; prednisolone tapered to 5 mg/day	16	F	Pancreas, kidneys (glomerulonephritis), parotid gland, eye (episcleritis), purpura legs	5	IgG4-RD/AIP1	No clinical improvement with IVMP. Partial response with cyclophosphamide. Significant improvement, no recurrence after 20 months with prednisolone plus rituximab	[14]
Case report	1	Prednisone; AZA	Prednisone; 50 mg/day for 9 months, dose reduction with addition of AZA (DNS)	13	F	Right-eye proptosis, Lacrimal gland	1	IgG4-related dacryoadenitis	Significant improvement with AZA, sustained clinical and radiological improvement	[30]
Case report	1	Surgery (bilateral submandibular gland excision)	NA	5	M	Bilateral submandibular glands	1	Chronic sclerosing sialadenitis IgG4-related	No complications post-surgery, no recurrence on follow-up	[31]
Case report	1	Prior steroid courses; surgery; prednisone	Prednisone 0.6 mg/kg tapered to 5 mg, then again at 10 mg/day	13	M	Orbit (mass), rectus muscle, pterygopalatine fossa, lymph nodes, liver, spleen	5	Probable/possible IgG4-RD	Initial improvement, relapse after tapering to 5 mg, stabilized with 10 mg/day	[32]
Case report	1	OP; prednisone plus AZA	Prednisone: 0.6 mg/kg/day as induction, tapered after 4 weeks to 5 mg/day over 3 months; prednisone 7.5 mg/day plus AZA 2 mg/kg/day started upon relapse 12 months later	7	F	Pancreas, liver (sclerosing cholangitis); lungs	3	IgG4-RD definite diffuse = type AIP, definite sclerosing cholangitis, probable lung involvement	Partial symptoms improvement after prednisone, but relapse occurred needing restart of steroids plus AZA. Patient under partial remission for 3 years	[33]
Case report	1	Surgery (excision of right neck mass and lymph node)	NA	13	F	Right cervical lymph nodes (PTGC; right neck mass)	1	IgG4-related LAD	Outcomes not reported	[34]
Case report	1	OP	Prednisone: 40 mg/day for 2 weeks, tapered over 4 weeks	16	M	Bilateral submandibular gland (enlargement)	1	IgG4-RD, chronic sialadenitis	Significant improvement in swelling after 1 year of follow-up	[35]
Case report	1	Whipple procedure, pancreatic enzyme replacement	NA	10	M	Pancreas (mass in pancreatic head), bile ducts (dilation)	2	AIP 1 (based on IgG4 plasma cells), AIP 2 (based on GELS)	No recurrence for 7 years, no autoimmune conditions	[36]
Case report	1	Stent placement; prednisone	Prednisone: 40 mg/day for 1 month, tapering 5 mg/week	15	M	Pancreas (mass in pancreatic head), biliary dilation	2	AIP 1	Resolution of stricture and shrinking of mass within 8 weeks of treatment. No recurrence for 13 months off steroids, developed celiac disease 1 year after diagnosis	[36]
Case report	1	Prednisone; then 6-mercaptopurine and amitriptyline	DNS	11	F	Pancreas (enlarged, ductal stricture, dilated pancreatic duct)	1	AIP 1	Relapse after prednisone. Symptoms controlled with 6-mercaptopurine, no recurrence of pancreatitis for 5 years; IgG4 levels remain high	[36]
Case report	1	MP; resection of stenotic fibers	MP: first systemic then oral reaching 8 mg/day for maintenance	17	F	Trachea (stenosis)	1	Hyper IgG4-RD	Significant improvement in tracheal patency, asymptomatic after 2 years but developed iatrogenic Cushing after long steroid treatment; oral steroids continued as maintenance	[37]
Case report	1	Prednisone	Prednisone: 20 mg/day (0.7 mg/kg/day) for 3 weeks, tapered over 2 months	8	F	Pancreas (chronic pancreatitis, pancreatic atrophy)	1	Probable AIP 1	Complete resolution of symptoms (diarrhea, weight loss, abdominal pain), IgG4 level reduction, remains asymptomatic after 6 months	[38]
Case report	1	Steroids; rituximab; pulse IVMP plus rituximab	Steroids: DNS, long tapering; rituximab: 1 g (750 mg/m^2^) IV, 2 doses, 2 weeks apart; high-dose IVMP plus rituximab at same doses as before upon relapse	7	F	Kidney (nephrotic syndrome), eye (exophthalmos, optic nerve), arm (soft tissue edema); 3rd nerve palsy	4	IgG4-related sclerosing disease	Almost complete remission after rituximab; relapse; complete resolution of symptoms observed after second round of rituximab treatment combined with steroids	[39]
Case report	1	Prednisolone; prednisolone plus AZA; octreotide; surgery	Prednisolone: 1 mg/kg; AZA (DNS); Octreotide (DNS)	16	M	Pleura, mediastinum (mass), mesenteric lymph nodes	3	IgG4-RD	Initial partial response, but persistent pleural effusion only improved after surgery	[40]
Case report	1	OP; MMF	Prednisone: DNS, for 2 months. MMF: dose unspecified, started upon prednisone relapse	14	F	Orbit (right eye; mass)	1	IgG4-ROD	Decrease in orbital mass size and marked decrease in swelling but relapse after 2 months. Complete remission with MMF. Follow-up date not specified	[41]
Case report	1	Partial surgical debulking	NA	10	F	Orbit (right eye; swelling, mild ptosis, proptosis)	1	IgG4-ROD	Complete remission up to 18 months of follow up	[41]
Case report	1	Colchicine, esomeprazole, prednisolone, AZA	DNS; maintenance with prednisolone 5 mg/day	7	F	Small-bowel mesentery (sclerosing mesenteritis), lymph nodes, pericardium	3	IgG4-related sclerosing mesenteritis	Complete remission after 6 months, no recurrence after 24-month follow-up	[42]
Case report	1	Prednisolone plus UDCA; added AZA, mesalazine	Prednisolone: 1 mg/kg/day at diagnosis, 1.5 mg/kg/day 1 month after, weaned over 6 months and maintained at 0.1 mg/kg/day; UDCA (10 mg/kg/day), AZA (0.5 mg/kg/day), and mesalazine (50 mg/kg/day) added 1 month after diagnosis and maintained.	3	M	Liver (sclerosing cholangitis), colon (ulcerative colitis), bile ducts	3	IgG4 sclerosing cholangitis	Significant improvement already after 1 year of treatment and patient remains stable after 2 years of treatment with further improvements in ALT, AST, and bilirubin	[43]
Case report	1	Mesalazine, AZA, hydrocortisone, prednisone, adalimumab	Mesalazine: 2 g/BID, discontinued as the causative drug for pancreatitis; AZA: 100 mg/day; hydrocortisone: 100 mg 4 times/day; prednisone: 10 mg/day for 6 months; adalimumab: initiated after steroid weaning (DNS)	16	M	Colon (IBD), liver (primary sclerosing cholangitis), bile ducts, pancreas, kidney (lesions)	5	AIP 1	No response to mesalazine and AZA. Rapid response to steroids but relapse after steroid tapering. Rapid resolution of symptoms with adalimumab and normal bowel and liver function tests. Patient remains on adalimumab	[44]
Case report	1	Prednisone, adalimumab off-label	Prednisone: 40 mg/day, 7 days, tapered within 3 months; adalimumab: 40 mg subcutaneous, biweekly	9	F	Orbit (right eye, lacrimal gland enlargement, proptosis, swelling, mass)	1	IgG4-ROD	Initial resolution of symptoms but relapse upon steroid tapering. Complete resolution with adalimumab, no relapse for 1 year of follow up	[45]
Case report	1	Albendazole, followed by praziquantel and prednisolone; pulse MP; prednisolone plus AZA	Prednisolone: 1 mg/kg/day plus AZA 2 mg/kg/day; DNS for other treatments	7	M	Orbit (pseudotumor, left eye)	1	IgG4-ROD	Relapse occurred after pulse prednisolone but significant improvement with prednisolone and AZA	[11]
Case report	1	Partial nephrectomy with regional lymph node dissection	NA	11	M	Kidney (right upper pole mass)	1	Probable IgG4-RD	No recurrence 5 years post-resection, continues to be managed for stage III chronic kidney disease	[46]
Case report	1	High-dose steroids and rituximab, surgical intervention (biopsies)	DNS	NR	F	Larynx, paratracheal tissues, upper mediastinum	3	IgG4-RD	Stabilization of disease for 18 months, reduction in laryngeal findings after surgical intervention, clinical resolution of symptoms after treatment.	[47]
Case report	1	Debulking; OP plus MMF	Prednisone (1 mg/kg) plus MMF (600 mg/m^2^) BID; prednisone weaned, MMF maintained	5	F	Orbit (proptosis, orbital mass), left periocular swelling	1	IgG4-RD	Significant improvement; reduced proptosis and swelling post-treatment. Left periorbital swelling recurred intermittently upon steroids cessation	[48]
Retrospective (Case 14)	1	Dexamethasone plus AZA (initial treatment); AZA; dexamethasone plus rituximab; rituximab	DNS; rituximab for maintenance every 6 months	17	M	Lung, lymph nodes, brain	3	IgG4-RD	No response to initial treatment. Partial response to dexamethasone and rituximab followed by relapse, remains on rituximab maintenance	[49]
Case report	1	Pancreaticoduodenectomy	NA	12	F	Duodenum (obstruction due to multiple ulcers)	1	Isolated duodenal IgG4-RD	Surgery performed, significant improvement, doing well at 1-month follow-up	[50]
Retrospective (Case 9)	1	Steroids	DNS	10	M	Liver (mass, hepatomegaly), lymph nodes	2	IgG4-RD	Partial resolution of liver mass and improvement in biochemical parameters	[51]
Observational	1	OPL, MTX	Prednisolone: 1 mg/kg/day, tapered over 5 months; MTX for 12 months	14	F	Orbit (unilateral swelling, proptosis, lateral rectus muscle inflammation)	1	Probable IgG4-RD	Clinical and radiological improvement, no relapse	[12]
Observational	1	OPL, MTX	Prednisolone: 1 mg/kg/day, tapered over 4.5 months; MTX for 23 months	13.6	M	Orbit (unilateral swelling), lacrimal gland	2	Probable IgG4-RD	Clinical and radiological response, regression in the right lacrimal gland and eyelid findings on follow-up MRI at 5 months of treatment; no relapse	[12]
Observational	1	None initially; pulse MP, prednisolone	Pulse MP: DNS Prednisolone: 1 mg/kg/day for 1.5 months, tapered and stopped	16	F	Orbit (unilateral swelling)	1	Possible IgG4-RD	Spontaneous regression, but relapse 38 months after diagnosis treated with pulse MP, followed by prednisolone, tapered and stopped (outcome not reported)	[12]
Observational	1	Pulse MP; OPL plus AZA	Pulse MP: DNS; OPL (DNS) plus AZA (1.2 mg/kg/day)	10	M	Orbit (unilateral swelling, proptosis, eyelid swelling)	1	Possible IgG4-RD	No relapse after treatment	[12]
Observational	1	MP	Scheduled to receive MP for 3 days, DNS	13.3	F	Orbit (unilateral swelling and eyelid tenderness, soft tissue mass); lungs (nodules)	2	Probable IgG4-RD	Lost to follow-up	[12]
Observational	1	OPL, AZA; rituximab plus pulse MP; radiotherapy; AZA/OPL	OP, AZA: 1.3 mg/kg/day; rituximab + pulse MP after first relapse, DNS; radiotherapy for 10 days; maintenance: low-dose (DNS) prednisolone (discontinued at 20 months) plus AZA (DNS); second-course radiotherapy; AZA/OPL: maintenance, DNS	9.3	F	Orbit (unilateral swelling, proptosis), 5th cranial nerve	2	Probable IgG4-RD	No symptoms regression with OPL/AZA, stable lesions. Relapse after 27 months of pulse MP plus rituximab. Relapse at 29 months after radiotherapy. Resistance to OPL, rituximab, and AZA treatments. Remission after second course of radiotherapy. Remains on AZA and OPL	[12]
Observational	1	OPL plus AZA, MTX	AZA: 1.2 mg/kg/day, prednisolone: 0.2 mg/kg/day	4.2	M	Mesenteric lymph nodes, mesenteric mass	1	Probable IgG4-RD	Clinical response and partial radiological response, no relapse; remains on treatment	[12]
Observational	1	Pulse MP, followed by OPL; AZAmesalazine	OPL: 0.6 mg/kg/day, tapered and discontinued at 13 months; AZA: 1.2 mg/kg/day; mesalazine: DNS	15.4	M	Colon (ulcerative colitis), lymph nodes (cervical, mediastinal, and mesenteric); pancreas (pancreatitis), lungs	4	Definite IgG4-RD	Clinical response, partial regression of hepatosplenomegaly; no lymphadenopathy and normal spirometry at 13 months. No relapse. Remains on treatment with AZA and mesalazine	[12]
Retrospective	1	Prednisolone (start and maintenance in >90%), surgery (n = 4); not specified for the adolescent patient	Prednisolone: median starting dose 40 mg/day (range 15–60 mg), median treatment duration 153 days (range 8–1402 days).	14.5	M	Biliary system, Liver	2	IgG4 sclerosing cholangitis	Partial or complete response occurred in 95% of subjects; relapse in 42%. Outcomes not specified for the adolescent patient	[52]
Case series	5	Rituximab (n = 1), rituximab + sirolimus (n = 1), ruxolitinib (n = 2), surgery (n = 1)	NS	Median 13.6	F(1) M(4)	Orbit, hip muscle, peripancreatic tissue (n = 3); lymph nodes (polylymphadenopathy), pulmonary, renal and hepatic foci, dacryoadenitis with oedema of the eyelids (n = 2)	3 (n = 3), 5 (n = 2)	IgG4-RD	Rituximab successful in 2 cases, JAK inhibitors (ruxolitinib) effective in localized cases, surgery positive for 1	[53]
Case report	1	Steroids, bone marrow transplant	Steroids (1 mg/kg/day), bone marrow transplant from HLA-identical donor	2	NR	Kidney (membranous glomerulopathy, TIN), bone marrow (failure)	2	IgG4 related kidney disease	No clinical response to steroids, bone marrow transplant performed	[54]
Case series	1	None (watch-and-wait)	NA	15.9	F	Laterocervical lymph nodes	1	IgG4-related LAD	Asymptomatic, no clinical evolution (3.3 years of follow-up)	[55]
Case series	1	Prednisone, sirolimus	Prednisone: 1 mg/kg/day, Sirolimus for ALPS	15.4	M	Laterocervical lymph nodes, immune system (ALPS)	1	IgG4-related LAD, ALPS	Initial complete response, recurrence after steroid withdrawal, stable with sirolimus	[55]
Case series	1	Chemotherapy(prednisone, vinblastine, cyclophosphamide)	According to national protocol for NLPHL	11.8	M	Laterocervical lymph nodes (PTGC), Hodgkin lymphoma	2	IgG4-related LAD	Complete remission (2.6 years of follow-up)	[55]
Case series	1	Steroids, immunoglobulins, MMF	Steroids (DNS), MMF	13.7	M	Inguinal lymph nodes	1	IgG4-related LAD	Autoimmune cytopenias, responsive to immunosuppressants (3.3 years of follow-up)	[55]
Case series	1	None (watch-and-wait)	NA	17.5	F	Laterocervical lymph nodes	1	IgG4-related LAD	Asymptomatic, no clinical evolution (0.2 years of follow-up)	[55]
Case series	1	None (watch-and-wait)	NA	11.4	F	Laterocervical lymph nodes	1	IgG4-related LAD	Asymptomatic, no clinical evolution (0.8 years of follow-up)	[55]
Case series	1	None (watch-and-wait)	NA	12.6	F	Submandibular lymph nodes	1	IgG4-related LAD	Asymptomatic, no clinical evolution (2.1 years of follow-up)	[55]
Case report	1	Prednisolone; surgical tumor removal; MP and cyclophosphamide, MMF plus HCQ and MTX	Prednisolone: 20 mg/day, tapered; MP and cyclophosphamide for 6 months (steroids tapered over 2 years); MMF (2 g/day), HCQ (200 mg/day), MTX (15 mg/week, orally); MTX (10 mg/week) plus MMF (1 g) plus HCQ (200 mg/day) for maintenance	8	F	Orbit (pseudotumor), kidney (glomerulonephritis), sinusitis, lungs, skin	5	IgG4-RD	Relapse of orbital mass post-surgical removal. Good clinical response with MP plus cyclophosphamide, stable remission with MMF and HCQ. Exacerbation of chronic sinusitis in the following years treated with MTX. Stable remission maintained under immunosuppressive therapy	[56]
Case report	1	Surgical resection; OPL; rituximab added upon further symptoms	Prednisolone: 1 mg/kg/day tapering after 10 weeks, to 6 mg/m^2^, continued at 4 mg/day for another 5 months; rituximab: 4 doses (375 mg/m^2^/week)	3	M	Left lung (mediastinal large B-cell lymphoma), pleural and pericardial effusion	2	IgG4-RD	Complete remission within 10 months of rituximab initiation, no signs of disease after follow-up at 27 months after therapy onset and 6 months after prednisolone cessation	[57]
Case report	1	None (wait-and-see)	NA	16	M	Liver (hepato-splenomegaly), mesenteric and abdominal lymph nodes	2	IgG4-related LAD	No treatment required, patient stable after 4 years of follow-up	[58]
Case report	1	None (wait-and-see)	NA	14	M	Bilateral submandibular lymph nodes	1	IgG4-related LAD	No treatment administered, spontaneous resolution and no relapse over 8 years of follow-up	[58]
Case report	1	Surgery only	NA	11	M	Right submandibular gland	1	Chronic sclerosing sialadenitis IgG4-related	No recurrence after surgical removal and 1 year of follow-up	[59]
Case report	1	Oral steroids	Steroids: 0.6 mg/kg	14	M	Eye (bilateral proptosis, extraocular muscles)	1	IgG4-ROD	Initial improvement, but lost to follow-up	[60]
Case series (Case 3)	1	Prednisolone plus AZA	Prednisolone (1 mg/kg/day) plus AZA (2 mg/kg/day); prednisolone (0.5 mg/kg/day) plus AZA (1 mg/kg/day) post relapse	7	M	Orbit (left, swelling and proptosis, orbital mass), lacrimal glands, soft tissue, rectus muscle	2	IgG4-ROD	Initial improvement, relapse after 9 months, retreated with steroids plus AZA (outcome not reported)	[13]
Case series (Case 6)	1	Pulse IVMP	IVMP: 500 mg for 3 days, then 500 mg as IV bolus every 3 weeks for 6 courses, then tapered	7	M	Orbit (proptosis), lacrimal gland	2	IgG4-ROD	Complete response with relapse upon discontinuation	[13]
Case report	1	OPL plus mesalazine; prednisolone plus AZA; infliximab; hydrocortisone; adalimumab	OPL (0.5 mg/kg/day, tapered) plus mesalazine (2 g BID); AZA (2.5 mg/kg/day) plus prednisolone; hydrocortisone (IV 100 mg/day); infliximab (5 mg/kg, IV, as rescue therapy, 3 doses, every 2 weeks); adalimumab (160/80 mg induction, 40 mg/week maintenance)	16	F	Colon, pancreas (autoimmune pancreatitis), bile ducts	3	IgG4-sclerosing disease	Initial response to prednisolone but relapse upon tapering. Initial response to AZA plus prednisolone but another relapse upon steroid tapering. No response to hydrocortisone. Initial rapid clinical improvement with infliximab but symptom recurrence already before the second dose. Complete remission with adalimumab, remains in remission 12 months later	[61]
Case report	1	Oral steroids	DNS, steroids tapered	12	M	Right eye (posterior scleritis, choroidal osteoma)	1	Possible IgG4-ROD	Vision stabilized with regular follow-up	[62]
Case report	1	Surgery (gross total resection)	NA	16	M	CNS (dural-based mass, hyperostosis)	1	IgG4-RD	Asymptomatic for 16 months after surgery, no recurrence	[63]
Case report	1	Prednisone plus AZA; prednisone plus cyclosporine; left nephrectomy; added MMF	Prednisone (1 mg/kg/day, tapered) plus AZA (1 mg/kg/day); prednisone (1 mg/kg/day) plus cyclosporine (5 mg/kg/day), added MMF (2 g/day)	7	M	Kidney (tumor), skin (vasculitis), eye (uveitis)	3	IgG4-RD	Nephrectomy for kidney tumor; uveitis improved with combination of prednisone, cyclosporine, and MMF	[64]
Case report	1	Budesonide; prednisolone plus AZA	Budesonide: 9 mg/day, 1 month; prednisolone (1 mg/kg/day, tapered over 4 months) plus AZA (1.5 mg/kg/day, maintenance at same dose)	16	M	Liver, pancreas	2	IgG4-related cholangitis	Relapse after budesonide tapering. Complete resolution of strictures and normalization of liver function tests after prednisolone + AZA. No relapse at the 5-month follow-up	[65]
Case series	4	Oral/IV steroids (n = 4), MTX plus rituximab (n = 2), MMF plus rituximab (n = 1)	DNS	Median: 13.2	F(3) M(1)	Eye (ocular disease) (n = 3); salivary glands (Sialadenitis) (n = 1)	1 (n = 4)		All in remission after rituximab (n = 3); 1 patient (treated with MMF/rituximab) was later diagnosed with Hodgkin’s lymphoma	[66]
Case report	1	Prednisone, MTX	Prednisone: 0.4 mg/kg/day, tapered and discontinued; MTX added in follow-up visits (DNS)	10.5	M	Bilateral submandibular salivary glands, cervical lymph nodes, hip joints, lungs	4	IgG4-RD	Significant improvement with prednisone, albeit with remaining inflammation in hip joint. Complete remission with the addition of MTX; no relapse after 12 months	[67]
Case report	1	Prednisolone; prednisolone plus MMF; rituximab added to the above	Prednisolone only (2 mg/kg/day, tapered over 4 months), then plus MMF (1200 mg/m^2^/day) for 3 months; rituximab added after 3 months of MMF, prednisolone stopped	14	F	Biceps muscle, lymph nodes, liver, spleen	4	IgG4-RD	Relapse upon prednisolone tapering, poor response to MMF. Significant improvement with rituximab, mass regression	[68]
Case report	1	Surgery (pterional craniotomy)	NA	16	M	Pituitary, dura mater, craniopharyngioma	2	Secondary IgG4-related hypophysitis	Headache resolved, vision not regained, no residual tumor on follow-up	[69]
Case report	1	Antibiotics only (Cefixime)	DNS	10	F	Parotid and submandibular salivary glands, lymph nodes, lacrimal glands, skin	5	IgG4-RD	Partial regression of lymph nodes, followed by relapse	[70]
Case report	1	Prednisolone	0.6 mg/kg/day for 4 weeks	15	M	Lungs (bronchiectasis, recurrent hemoptysis)	1	Pulmonary IgG4-RD	Hemoptysis resolved, IgG4 levels declined; no relapse	[71]
Case series	1	Prednisolone	DNS	15	F	Sinonasal region (nasal septum, inferior turbinate, maxillary sinus)	1	IgG4-RD	Not reported; patient on follow-up	[72]
Case series	1	Prednisolone; rituximab	Prednisolone: 1 mg/kg/day; rituximab (two IV infusions of 600 mg, 15 days apart)	15	F	Sinonasal region (nasal cavity, maxillary and cavernous sinus), orbit (proptosis), retroperitoneum (fibrosis)	3	IgG4 sclerosing disease	Partial response to prednisolone, further improvement with rituximab, still under follow-up	[72]
Case report	1	Oral glucocorticoids plus cyclosporine, prednisone and IVIG; pulse MP, recombinant IFNb-1b; teriflunomide; MMF	Glucocorticoids plus cyclosporine (stopped after 3 months); prednisone and IVIG; MP 1 g for 5 days, followed by prednisone 70 mg/day (tapered); recombinant IFN b-1b every 2 days for 6 months, subcutaneously; teriflunomide 14 mg daily; OP 40 mg/day with MMF 750 mg BID	14	F	Eye (eyelid ptosis), lacrimal glands, brain parenchyma, spinal cord, lymph nodes	5	IgG4-RD	Improvement in eye ptosis and diplopia with prednisone plus IVIG. No relapse for over a year after prednisone tapering and administration of IFNb-1b. No response to teriflunomide for lymph node enlargement; Good response of lymphadenopathy to prednisone plus MMF. At the 7-month follow up: numbness/weakness resolved, intracranial lesions shrank	[73]
Retrospective case series	1	OPL (possible AZA as maintenance, not specified for which patients)	OPL: 1–2 mg/kg/day, subsequently tapered	10	M	Liver (mass)	1	IgG4 related hepatic mass	Good clinical response	[74]
Retrospective case series	1	OPL (possible AZA as maintenance, not specified for which patients)	OPL: 1–2 mg/kg/day, subsequently tapered	12	F	Abdomen (mass)	1	IgG4-RD	Good clinical response	[74]
Retrospective case series	1	OPL (possible AZA as maintenance, not specified for which patients)	OPL: 1–2 mg/kg/day, subsequently tapered	7	F	Kidney	1	IgG4-related TIN	Good clinical response	[74]
Retrospective case series	1	OPL (possible AZA as maintenance, not specified for which patients)	OPL: 1–2 mg/kg/day, subsequently tapered	14	M	Right eye (proptosis, orbital mass), lacrimal gland	2	IgG4-RD	Good clinical response	[74]
Case report	1	OPL, MMF	OPL: 50 mg/day for 1 month, tapered and restarted after relapse; retapering at 5 mg/day. MMF as SSA: 300 mg/m^2^ BID	9	F	Eye (bipalpebral volume increase, right predominance exophthalmos) lacrimal gland	2	IgG4-related ophthalmic disease	Initial response to prednisone but relapse after tapering. Prednisone restarted and Cushing syndrome developed at the 3rd month of treatment. MMF allowed prednisone tapering to 5 mg/day and disease control up to the 5-month follow-up	[75]
Case report	1	Prednisone	Prednisone: 30 mg/day (0.5 mg/kg/day), tapered over 3 months	17	M	Liver, bile ducts (multiple intrahepatic strictures)	2	IgG4 sclerosing cholangitis	Improvement in liver enzymes and symptoms after 1 month, stable after prednisone weaning	[76]
Case report	1	Prednisone plus AZA; MMF plus steroids, pulse MP plus rituximab; rituximab maintenance dose plus prednisolone	Prednisone 0.6 mg/kg/day plus AZA 2.5 mg/kg/day; MMF DNS; rituximab: 500 mg IV, 4 doses, once weekly, maintenance dose 500 mg once plus prednisolone 1 mg/kg	13	F	Pericardium, pleura, lungs	3	IgG4-RD	Initial improvement with prednisone plus AZA and remained symptom free for 2 years with MMF; marked initial improvement with rituximab but relapsed again after 6 months; died from massive hemoptysis after relapse	[77]
Retrospective (Case 6)	1	Oral steroids; not specified for the pediatric patient	1 mg/kg/day	8	F	Submandibular salivary glands	1	IgG4-RD	Response to steroids in most cases; not specified for the pediatric patient	[78]
Case report	1	OP; pulse IVMP; rituximab	OP: 40 mg/day, tapered to 10 mg in 1 month; IVMP: 250 mg every 6 h for 3 days; rituximab: 1 g IV, 2 doses 2 weeks apart	12	F	Right orbit (proptosis, orbital mass), left oculomotor nerve, sinuses, cavernous sinus	3	IgG4-related sclerosing disease	Initial improvement with OP but relapse occurred during tapering. Minimal clinical improvement with IVMP. Complete resolution of ptosis and normalization of eye movement with rituximab; no relapse up to 1 year of follow-up	[10]
Case report	1	Surgery, no further treatment	NA	16	F	Skin (pseudolymphoma), thighs, abdominal wall	3	IgG4-RD	No recurrence after surgical removal and follow-up	[79]
Case report	1	Corticosteroids; corticosteroids plus MMF	Corticosteroids (initial DNS, discontinued after 6 weeks), MMF plus steroids (discontinued after 4 months); MMF for maintenance	17	F	Right optic disc, CNS (pachymeningitis, dural thickening [frontotemporal regions]), 6th nerve palsy	3	IgG4-RD	Initial resolution of ocular symptoms with corticosteroids but relapse after 4 months. Complete remission after MMF plus steroids, no relapse after 2 years of follow-up	[80]
Case report	1	Surgical excision	NA	7	M	Lung (IPT)	1	IgG4-RD	Symptom-free post-surgery with limited fibrotic changes	[81]
Case report	1	OPL, MTX plus TGF-b2 (Modulen)	OPL: 2 mg/kg/day, slow tapering for >4 months; MTX 12.7 mg/m^2^ (discontinued after 3 months) plus Modulen 2.5–3 L/day for 8 weeks	16	F	Left eye (orbital inflammation), colon (colitis)	2	IgG4-RD	Improvement in ophthalmic symptoms with prednisolone but disease progression after 9 months with intestinal involvement. Significant improvement in symptoms and weight gain. Patient lost to follow-up	[82]
Case report	1	Antibiotics (ceftriaxone, then cefixime and azithromycin); surgery; cefpodoxime plus betamethasone; cephalosporins and corticosteroids	Antibiotic courses followed by partial resection of parotid gland, followed by another antibiotic plus betamethasone—DNS	6	M	Parotid gland (swelling, recurrent parotitis), lymph nodes	2	Chronic sclerosing sialadenitis IgG4-related	Good response to antibiotics and steroids; no relapse at 2-month follow up	[83]
Case report	1	Prednisone; added AZA and antibiotic prophylaxis (trimethoprim/sulfamethoxazole); MMF	Prednisone: 1 mg/kg/day for 3 weeks, then tapered; AZA and MMF: DNS	1.25	M	Left orbit (medial and inferior; mass), rectus muscle	2	IgG4-ROD	Orbital disease settled at 1-year follow-up, maintained on low-dose prednisone and MMF	[84]
Case report	1	Pulse IVMP; AZA	IVMP: 1 g/day for 5 days; AZA: 2 mg/kg/day, 2 months later; IV glucocorticoids administered again 2 years later upon relapse and stopped, AZA continued	17	F	Spinal cord (longitudinally extensive transverse myelitis), pituitary (asymptomatic hypophysitis)	2	Possible IgG4-related hypophysitis	Significant improvement, relapse 2 years after initial treatment, stable remission with AZA up to 5 years follow-up	[85]
Case report	1	Corticosteroid (Deflazacort)	Equivalent to 1 mg/kg/day prednisolone for 7 months	13	M	Mediastinal lymph nodes, thymus (hyperplasia), heart (coronary artery aneurysm, aortitis, pericardial effusion)	4	IgG4-RD	ESR and CRP normalized, IgG4 decreased to 143 mg/dL, coronary artery size returned to normal on cardiac MRI, no relapse at 1-year follow-up	[86]
Case report	1	Prednisone and MMF	Prednisone: 1 mg/kg/day; MMF: 0.25 g/day; Treatment lasted over 8 months	3	M	Liver (hepatic IPT)	1	IgG4-related hepatic IPT	Reduction in hepatic mass and improvement in anemia	[87]
Case report	1	Craniotomy (tumor removal); pulse MP, rituximab, cyclophosphamide; prednisolone	MP (500 mg/day for 3 days), rituximab (500 mg IV every 2 weeks, 2 doses), cyclophosphamide (500 mg IV/month, 3 doses); prednisolone 10 mg/day for maintenance	17	F	Brain (cerebral pseudotumor), trigeminal nerve (perineural spreading), optic nerve (compression), bilateral submandibular gland, lymph nodes (neck, right side)	5	IgG4-related cerebral pseudotumor with compressive optic neuropathy	Significant visual improvement; MRI showed almost complete disappearance of the lesions after immunosuppressive therapy	[88]

Notes: Articles are listed in alphabetic order according to the first author’s surname. From all studies listed, only patients younger than 18 years were selected, and their information is presented in the table. In grey highlight, we mark studies including more than one patient, who are listed in separate rows for display purposes. Abbreviations: ALPS, autoimmune lymphoproliferative syndrome; AIP, autoimmune pancreatitis; BID, twice daily; CNS, central nervous system; DNS, dose(s) not specified; HCQ, hydroxychloroquine; HLA, human leukocyte antigen; IBD, inflammatory bowel disease; IPT, inflammatory pseudotumor; IVMP, intravenous methylprednisolone; LAD, lymphadenopathy; MMF, mycophenolate mofetil; MP, methylprednisolone; MTX, methotrexate; NA, not applicable; NLPHL, nodular lymphocyte-predominant Hodgkin lymphoma; OP, oral prednisone; OPL, oral prednisolone; PET/CT, positron emission tomography/computed tomography; PTGC, progressive transformation of germinal centers; RD, related disease; ROD, related orbital/ophthalmic disease; SSA, steroid-sparing agent; TIN, tubular interstitial nephritis; UDCA, ursodeoxycholic acid.

## Data Availability

No new data were created or analyzed in this study. Data sharing is not applicable to this article.
